# A Rare Case of Synchronous Invasive Adenocarcinoma of the Colon and Marginal Zone Lymphoma of a Splenule Associated With Hemolytic Anemia

**DOI:** 10.7759/cureus.55843

**Published:** 2024-03-09

**Authors:** Debduti Mukhopadhyay, Taher Sbitli, Anandita Kishore, Elijah R Ilasin, Umair Masood

**Affiliations:** 1 Department of Internal Medicine, University at Buffalo Jacobs School of Medicine and Biomedical Sciences, Buffalo, USA; 2 Department of Sciences, University of Western Ontario, London, CAN; 3 Department of Gastroenterology, Mercy Hospital of Buffalo, Buffalo, USA

**Keywords:** marginal zone lymphoma (mzl), lymphoma, colon cancer, hemolytic anemia, adenocarcinoma

## Abstract

This case report presents a rare and intriguing clinical scenario involving a 63-year-old male with recurrent left-sided hydroureteronephrosis, hypertension, and hyperlipidemia presenting with fatigue, dyspnea, and weight loss. Laboratory findings revealed anemia, basophilic stippling, spherocytosis, and nucleated red blood cells on the peripheral blood smear, raising concerns for hemolysis. Concomitant iron deficiency anemia led to further investigations, revealing gastritis and a colonic mass. A CT scan revealed splenomegaly with an accessory spleen. The histopathological evaluation identified splenic marginal zone lymphoma (MZL) - a diagnosis supported by flow cytometry. Simultaneously, the patient was found to have a moderately differentiated colorectal adenocarcinoma on colonoscopy. This unique case highlights a rare synchronous occurrence of invasive colonic adenocarcinoma with splenule MZL, an unprecedented finding in medical literature.

## Introduction

Initially described in 1899 by Billroth and subsequently defined in 1932 by Warren and Gates, multiple primary malignancies (MPM) are classified as either synchronous (tumors diagnosed at the same time) or metachronous (the second tumor detected six months after the initial diagnosis) [1,2]. This case involves a 63-year-old Caucasian male presenting with fatigue, dyspnea, and weight loss, diagnosed with synchronous primary adenocarcinoma of the colon and splenule marginal zone lymphoma (MZL). An esophagogastroduodenoscopy and colonoscopy revealed gastritis and a colon mass that was confirmed through biopsy to be an invasive adenocarcinoma. A CT scan showed transverse colon thickening and splenomegaly with an accessory spleen. A laparoscopic hemicolectomy with splenectomy revealed splenic MZL. To our knowledge, our case is the first reported instance of invasive colonic adenocarcinoma with synchronous splenule MZL.

This case was presented as a poster at the Lymphoma, Leukemia, and Myeloma Congress in NYC on October 18, 2023.

## Case presentation

A 63-year-old Caucasian male, with a history of recurrent left-sided hydroureteronephrosis due to calculus, hypertension, and hyperlipidemia, presented with progressive fatigue, shortness of breath, and an unintentional weight loss of 25 pounds. Laboratory results were significant for a hemoglobin level of 5.3 g/dL, leukopenia, and borderline macrocytosis. Additionally, his iron saturation was low at 17%, total bilirubin was elevated at 1.9 mg/dL, Lactate Dehydrogenase (LDH) was high at 249 U/L, and haptoglobin was low at less than 30 mg/dL, indicating hemolysis. The peripheral blood smear revealed basophilic stippling, spherocytosis, and nucleated red blood cells.

**Figure 1:**
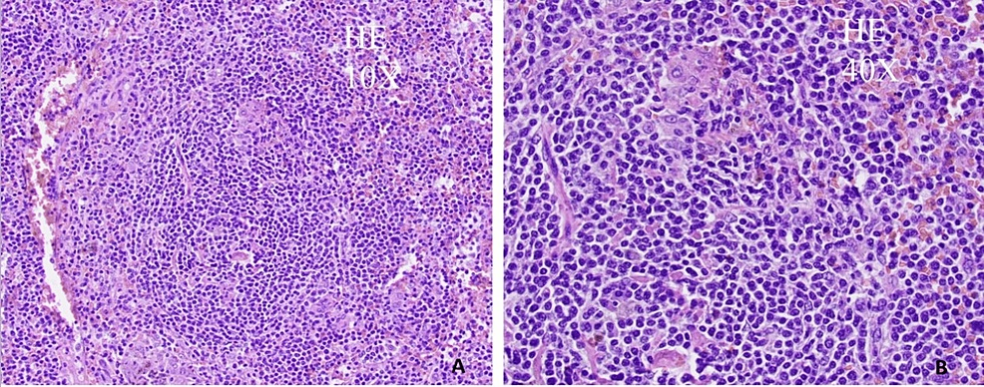
There is nodular lymphoid infiltrate extensively involving white pulp and extending to red pulp. The lymphoid infiltrate is composed of mostly small-sized lymphoid cells with slightly irregular nuclear contours, condensed nuclear chromatin, and inconspicuous nucleoli. No large cell components seen.

**Figure 2:**
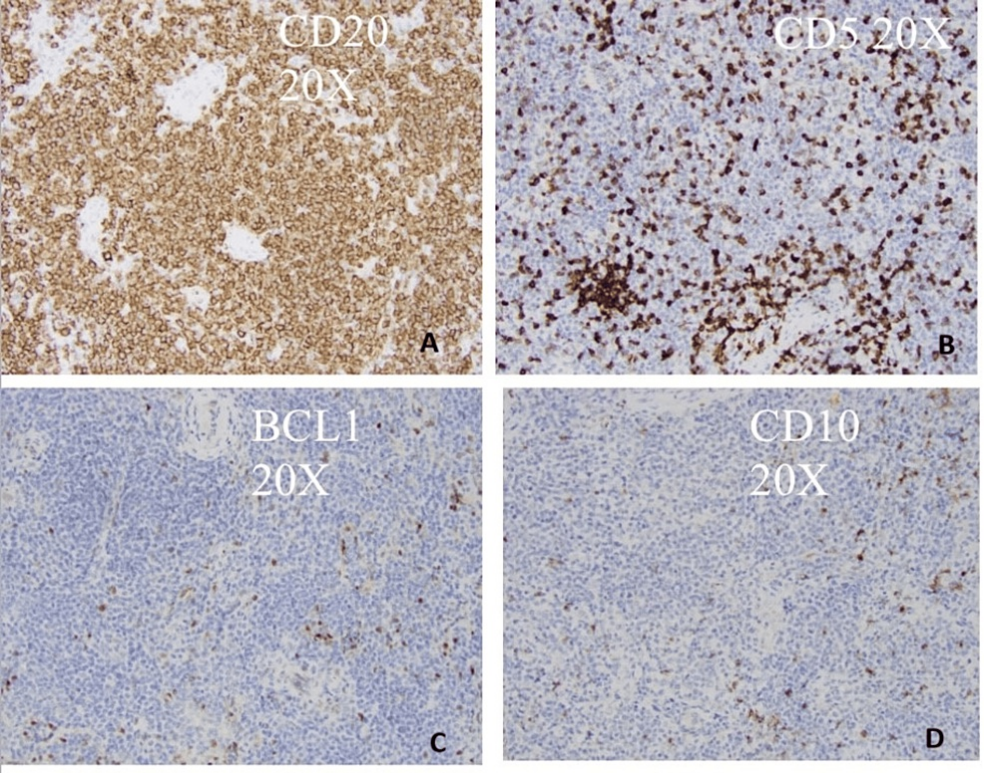
The lymphoma cells are positive for CD20 but negative for CD5, CD10, and BCL1.

Given the suspicion of blood loss anemia, the patient underwent an esophagogastroduodenoscopy, revealing gastritis and gastric compression from an external source. A subsequent colonoscopy revealed a 2.5 cm mass in the descending colon. Biopsies from the descending colon revealed invasive moderately differentiated colonic adenocarcinoma. A contrast-enhanced computer tomography (CT) scan of the abdomen and pelvis showed mild circumferential wall thickening in the transverse colon without a discrete mass. Splenomegaly with the presence of a splenule was also identified. The patient underwent a laparoscopic transverse hemicolectomy, and the accessory spleen was removed. Flow cytometry and histopathological evaluation of the splenic specimen detected a mature B-cell lymphoproliferative neoplasm expressing CD45 and lambda light chain. CD5, CD10, CD38, BCL1, SOX11, and CD43 testing were negative, while positive for CD20, CD19, PAX5, and BCL2 (Figures 1A, 1B, 2A-2D). The Ki-67 proliferation index was approximately 40%, leading to a diagnosis of splenic MZL.

Although the bone marrow biopsy did not yield a conclusive diagnosis, the lymphoma cell phenotype in the bone marrow resembled that of the splenic MZL. The patient was initiated on a combination of rituximab and bendamustine. The final pathology report of the colon specimen revealed moderately differentiated colorectal adenocarcinoma at stage pT2N0 with microsatellite stability (MSS), marked by tumor budding, angiolymphatic invasion, and perineural invasion. A chest CT for staging revealed subtle areas of pulmonary stranding and nodularity within the right middle lobe and left upper lobe, possibly related to an infectious/inflammatory process and unlikely to be metastatic. While admitted the patient received multiple blood transfusions to mitigate the anemia. We concluded that the cause of his anemia was multifactorial including B-cell clonal disorder leading to hemolysis and colonic adenocarcinoma leading to iron deficiency. The patient was advised to undergo CT scans every six months and a colonoscopy after one year.

## Discussion

Although initially defined by Warren and Gates as either synchronous or metachronous, MPM definitions vary across studies. For instance, the Surveillance, Epidemiology, and End Results (SEER) project defines synchronous tumors as two or more primary malignancies diagnosed within two months, whereas the International Association of Cancer Registries and the International Agency for Research on Cancer (IACR/IARC) uses a six-month time frame for the same criterion [3].

The diagnosis of MPM, while uncommon, has seen an uptick in recent years. This trend is likely due to advancements in imaging techniques and improved survival rates resulting from modern therapeutic interventions. The incidence of MPM is estimated to range from 0.52% to 11.7% [4]. Gastrointestinal malignancies account for the majority of MPM cases at 39.1%, followed by urogenital and respiratory malignancies at 24.5% and 17.1%, respectively [4]. Hematologic and lymphatic malignancies make up 8.1% of cases [4]. The most commonly associated synchronous lymphoma with adenocarcinoma is Diffuse large B-cell lymphoma [5]. To our knowledge, our case is the first reported instance of invasive colonic adenocarcinoma with synchronous splenule MZL. There have been reports of a case involving rectal adenocarcinoma with splenic lymph node MZL [5].

MZL is a rather rare pathology with incidence of 0.13 per 100,000 persons per year with a median age at diagnosis of 69 years [6]. With an indolent course and heterogeneously hard-to-predict prognostic factors, MZL carries a median survival of eight to 10 years with a large portion of patients ~30% experiencing a more complicated course and worse outcomes [7]. Presentations are variable and usually mount to autoimmune manifestations including autoimmune hemolytic anemia, immune thrombocytopenia, and cold agglutinin disease among others. Diagnosis is based on findings on splenic histopathology. immunohistochemistry antibodies staining should be utilized to recognize MZL as well as exclude other disorders. MZL often expresses CD20, CD79a, BCL2, and surface IgM [7]. There is no consensus on gold standard therapy but rather on tailoring the management based on clinical symptoms. Options include splenectomy and chemotherapy in combination with rituximab or rituximab alone.

The pathophysiologic mechanism underlying the synchronous development of solid tumors and lymphomas is unclear. Proposed mechanisms include obstructed lymphatic channels which may favor the development of solid tumors, suppression of lymphocytic apoptotic antitumor responses, and chronic inflammation generated by solid tumors triggering a lymphoma [5].

## Conclusions

In conclusion, the synchronous development of MPM is an uprising occurrence. A high index of suspicion to achieve an early diagnosis is vital. As in our case, presentations can be non-specific. This case highlights the evolving landscape of cancer diagnosis influenced by advances in imaging and therapeutic interventions. The unclear pathophysiology linking synchronous tumors necessitates further exploration. As these cases become more frequent, an understanding of these mechanisms becomes crucial to enhance treatment strategies and improve outcomes.
